# Human methylome variation across Infinium 450K data on the Gene Expression Omnibus

**DOI:** 10.1093/nargab/lqab025

**Published:** 2021-04-22

**Authors:** Sean K Maden, Reid F Thompson, Kasper D Hansen, Abhinav Nellore

**Affiliations:** Computational Biology Program, Oregon Health & Science University, Portland, OR 97239, USA; Department of Biomedical Engineering, Oregon Health & Science University, Portland, OR 97239, USA; Computational Biology Program, Oregon Health & Science University, Portland, OR 97239, USA; Department of Biomedical Engineering, Oregon Health & Science University, Portland, OR 97239, USA; VA Portland Healthcare System, Portland, OR 97239, USA; Department of Medical Informatics & Clinical Epidemiology, Oregon Health & Science University, Portland, OR 97239, USA; Department of Radiation Medicine, Oregon Health & Science University, Portland, OR 97239, USA; Department of Genetic Medicine, Johns Hopkins University School of Medicine, Baltimore, MD 21287, USA; Department of Biostatistics, Johns Hopkins Bloomberg School of Public Health, Baltimore, MD 21205, USA; Computational Biology Program, Oregon Health & Science University, Portland, OR 97239, USA; Department of Biomedical Engineering, Oregon Health & Science University, Portland, OR 97239, USA; Department of Surgery, Oregon Health & Science University, Portland, OR 97239, USA

## Abstract

While DNA methylation (DNAm) is the most-studied epigenetic mark, few recent studies probe the breadth of publicly available DNAm array samples. We collectively analyzed 35 360 Illumina Infinium HumanMethylation450K DNAm array samples published on the Gene Expression Omnibus. We learned a controlled vocabulary of sample labels by applying regular expressions to metadata and used existing models to predict various sample properties including epigenetic age. We found approximately two-thirds of samples were from blood, one-quarter were from brain and one-third were from cancer patients. About 19% of samples failed at least one of Illumina’s 17 prescribed quality assessments; signal distributions across samples suggest modifying manufacturer-recommended thresholds for failure would make these assessments more informative. We further analyzed DNAm variances in seven tissues (adipose, nasal, blood, brain, buccal, sperm and liver) and characterized specific probes distinguishing them. Finally, we compiled DNAm array data and metadata, including our learned and predicted sample labels, into database files accessible via the recountmethylation R/Bioconductor companion package. Its vignettes walk the user through some analyses contained in this paper.

## INTRODUCTION

DNA methylation (DNAm, Table 1) has been widely studied for its roles in normal tissue development (1–4), biological aging (5–7) and disease (8–12). DNAm regulates gene expression, either in *cis* if it occurs in a gene’s promoter, or in *trans* if it overlaps an enhancer or insulator (4,9,13). Whole-genome DNAm (or ‘methylome’) analysis, especially in epigenome-wide association studies (EWAS), is a common strategy to identify epigenetic biomarkers with potential for clinical applications such as in prognostic or diagnostic panels (14–16).

**Table 1.** Abbreviations used frequently in this paper

Term	Meaning	Description
GEO	Gene Expression Omnibus	Public database containing all methylation array data analyzed in this paper.
GSE	Study accession number	Unique identifier for a study record in GEO that includes a platform, set of sample records and supplemental matrices containing assay data.
GSM	Sample accession number	Unique identifier for a sample record that includes sample-specific metadata and may also include supplemental sample datasets.
CpG	Cytosine-guanine dinucleotide	Dinucleotide sequence, or locus, consisting of a cytosine followed by a guanine.
DNAm	DNA methylation	The presence of a nucleotide-bound methyl group, typically at the 5’ cytosine position in a CpG locus.
HM450K	HumanMethylome 450K	Popular array platform, manufactured by Illumina, that uses BeadArray technology to probe DNAm at roughly 480 000 CpG loci.

Most investigations probe DNAm with array-based platforms. Published DNAm array data and sample metadata are commonly available through several public resources. These include cross-study databases like the Gene Expression Omnibus (GEO) (17,18) and ArrayExpress (19), as well as landmark consortium studies like the Cancer Genome Atlas (TCGA) (20) and the Encyclopedia of DNA Elements (ENCODE) (21,22). Recently published databases and interfaces provide access to samples from these sources (23–27).

While over 1604 DNAm array studies and over 104 000 samples have been submitted to GEO since 2009 (Supplementary Figure S1), there have been few attempts to rigorously characterize technical and biological variation across these studies. In 2013, two studies independently compiled DNAm array samples from GEO and elsewhere, analyzing epigenetic age across tissues and diseases (5), and investigating cross-study normalization (28). More recent cross-study analyses include (29) from 2018, which evaluated metadata and sample quality across 8327 DNAm array samples, and (30) from 2020, which validated sperm-specific DNAm patterns using 6288 samples.

While the GEO website provides access to submitted experiment and sample metadata, the metadata are not necessarily structured and require harmonization to facilitate cross-study analyses. There are currently no R/Bioconductor (31) packages providing access to uniformly normalized array data across GEO studies accompanied by harmonized metadata. It should also be noted that most GEO studies do not include raw intensity data (IDAT) files, which are needed to uniformly normalize samples and thus limits their utility for novel cross-study analyses.

The vast majority of GEO DNAm array data is composed of samples using Illumina’s HumanMethylation 450K (HM450K) BeadArray platform. Restricting attention to HM450K samples with IDATs published on or before 31 March 2019, we identified 35 360 samples from 362 studies, over three times the number of samples studied by either (5),(28), or (29). From sample IDATs, we extracted raw signals and probe significance data, derived quality metrics from control probe data and performed normalization on out-of-band signal with the noob method (32). We also learned a controlled vocabulary of sample labels by applying regular expressions to metadata and used existing DNAm array-based models to predict sex, epigenetic age and blood cell fractions (5,33,34). We conducted analyses investigating the performances of standard quality assessments and identified studies with frequent failed samples. Finally, we characterized autosomal DNAm variation in 7484 samples from seven non-cancer tissue types. This analysis complements recent independent efforts to quantify tissue-specific DNAm patterns (30) and showcases several of the relatively rare sample types we compiled from GEO (e.g. sperm, adipose and nasal).

To aid other investigators interested in reanalyzing DNAm array data from GEO, we compiled raw and noob-normalized DNAm array data with our learned and predicted metadata into Hierarchical Data Format 5 (HDF5)-based databases accessible using recountmethylation, a companion R/Bioconductor (31) package available at https://doi.org/doi:10.18129/B9.bioc.recountmethylation. Use of this package is covered thoroughly in accompanying vignettes, which also reproduce some of the results contained in this paper.

## MATERIALS AND METHODS

### Discovery and download of DNAm array IDATs on GEO

We used the esearch function of Entrez Programming Utilities v10.9 to search for every HM450K sample published to GEO as of 31 March 2019 for which two gzip-compressed IDAT download URLs were available. We ultimately downloaded IDATs for 35,360 sample records. Search and download were performed using the script https://github.com/metamaden/recountmethylation_server/blob/master/src/server.py. Note, the HM450K platform accession ID, GPL13534, is specified in the script https://github.com/metamaden/recountmethylation_server/blob/master/src/settings.py, and changing this will cause the server to target a different array platform.

### Preprocessing of DNAm array IDATs on GEO

We preprocessed DNAm array IDAT pairs for 35 360 HM450K samples on GEO using the R/Bioconductor package minfi v1.29.3 (33), applying the normalized exponential out-of-band probe method (i.e. noob normalization) in the analysis pipeline at https://github.com/metamaden/recountmethylation.pipeline. The noob normalization technique mitigates run-specific technical biases and precedes batch- and/or study-level normalization steps (32).

### Quality control results

We computed 19 quality metrics from red and green color channel signals for HM450K samples (Supplementary Table S2). To obtain the 17 BeadArray controls, we referred to Illumina’s official documentation (35,36) as well as methods in the ewastools v1.7 package (29). For our calculations, we used signal from the extension green control as background, and we used a denominator offset of 1 where it would otherwise be 0 (Supplemental Information) (29)). These calculations were done with the script https://github.com/metamaden/recountmethylationManuscriptSupplement/blob/main/R/beadarray_cgctrlmetrics.R. We thereby obtained a binary matrix of outcomes across the 17 BeadArray controls, where pass = 1, and fail = 0, on which we performed PCA using the ‘prcomp’ R function from the stats v4.0.2 R package. We then used ANOVAs to determine the variances explained by each control for each component, and we obtained stacked barplots of component variances with ggplot2 (Supplementary Figure S4). The script https://github.com/metamaden/recountmethylationManuscriptSupplement/blob/main/inst/scripts/figures/figS4.R reproduces our steps.

We subsequently computed array-wide *log*_2_ median methylated and *log*_2_ median unmethylated signals, as reproduced in the recountmethylation data analysis vignette at https://www.bioconductor.org/packages/release/bioc/vignettes/recountmethylation/inst/doc/recountmethylation_data_analyses.pdf.

### Obtaining sample metadata

Sample metadata was downloaded from GEO as study-level Simple Omnibus Format in Text (SOFT) files using the script https://github.com/metamaden/recountmethylation_server/blob/master/src/dl.py. From SOFT files, sample-level metadata were extracted as JSON-formatted files. Study-specific metadata fields were filtered prior to learning sample annotations (below). These steps were performed using the scripts at https://github.com/metamaden/recountmethylationManuscriptSupplement/tree/main/inst/scripts/metadata.

### Learning sample annotations

We took a partially automated approach to learn sample annotations from mined metadata (Supplementary Table S1). Our annotations were inspired by those in Marmal-aid (28) and included disease/experiment group, age and sex (Supplementary Table S1, (28)). To learn labels, we first coerced SOFT-derived metadata into annotation terms, then used manually constructed regular expressions to extract new labels (Supplemental Information).

### Learning sample type predictions

We learned additional metadata using the MetaSRA-pipeline (https://github.com/deweylab/MetaSRA-pipeline (37), Supplementary Table S1, (38)). This pipeline uses natural language processing to map sample metadata to curated ontology terms from the ENCODE project. It returns mapped terms and sample type confidences for each of six categories. We retained categories with the highest-confidence predictions as the most-likely sample types (Supplementary Table S1, Figure S2 and Supplemental Information).

### Model-based metadata predictions from DNAm

After noob normalization, we performed model-based predictions of sample age (5), sex (33) and blood cell type fractions (34) using the minfi (v.1.29.3) and wateRmelon (v.1.28.0) R/Bioconductor packages in our script https://github.com/metamaden/recountmethylationManuscriptSupplement/blob/main/inst/scripts/metadata/metadata_model_predictions.R. We tested concordance of mined and predicted sex and age to inform the use of these predictions and reliability of learned annotations (Results).

### Principal component analyses of autosomal DNAm

We performed array-wide approximate principal component analyses (PCA) with the stats (v.3.6.0) R package, using noob-normalized autosomal DNAm from all samples and a subset of filtered samples from seven non-cancer tissues (Beta-values, Figure 3 and Supplementary Figure S6). Missing values were imputed by array-wide DNAm medians (Beta-value scale) within samples. To improve computational efficiency, we first applied feature hashing (also known as the hashing trick) (39,40) to project the normalized Beta-value arrays into an intermediate reduced space before performing PCA. PCA results were visually almost identical whether we invoked an intermediate dimension of 1000 or 10 000 (results not shown). We used the 1000-dimension mapping for analyses in Figure 3 (data provided in Supplementary Files). The above analysis steps are shown in the script https://github.com/metamaden/recountmethylationManuscriptSupplement/blob/main/inst/scripts/analyses/pca_analysis_fig3.R.

### Annotation of studies for cross-tissue DNAm variability analyses

We identified samples of seven distinct tissues (adipose, blood, brain, buccal, liver, nasal and sperm), where each tissue included at least 100 samples across at least two study records (Supplementary Table S6). While we noted sufficient samples from placenta (study accessions GSE100197,GSE71678 and GSE74738), these were omitted due to high differences between mined and predicted ages, which prevented imputation using epigenetic age as for other tissues (below). We summarized study characteristics, including phenotype or disease of interest, in Supplementary Table S6. Targeted samples were from a variety of studies targeting various diseases, syndromes, disorders and exposures. Patient demographics spanned all life stages, including fetal, infant, child and young and old adult, and several studies focused on ethnic groups not commonly studied (e.g. Gambian children from record GSE100563;GSE100561).

### Preprocessing and analyzing seven non-cancer tissues for DNAm variability analyses

We studied samples in seven tissue types, including adipose, blood, brain, buccal, nasal, liver and sperm (Supplementary Table S6, Supplementary Figures S7a and S7b). We removed likely low-quality samples that showed low study-specific (less than fifth quantile) methylated and unmethylated signal, or showed signal below manufacturer-prescribed quality thresholds for at least one BeadArray control. We also removed putative replicates according to genotype-based identity predictions from ewastools (Supplemental Information, (29)).

We preprocessed noob-normalized DNAm for each tissue separately. First, we performed linear model adjustment on study IDs using DNAm *M*-values, defined as *logit*(Beta), with the limma v3.39.12 package. We then converted the adjusted DNAm to Beta-value scale. To account for the impact of confounding variables, we removed probes whose DNAm variances showed significant (p-adjusted < 0.01) and substantial (percent variance ≥ 10%) contributions from model-based predictions of age, sex and cell type fractions, which removed 39 000 to 194 000 (8–40% of) probes across tissues (ANOVAs, Supplementary Figure S7c).

After preprocessing, we identified probes with recurrent low variance and low mean intervals (max–min, mean tissue-wise DNAm, <0.01 or 1%) across seven distinct tissues. We also identified probes with high and tissue-specific variance. For each analysis we used a two-step probe selection process in each tissue where we selected (i) probes in the highest or lowest 10th quantile of variance (e.g. an absolute quantile variance filter), and (ii) probes in the highest or lowest 10th quantile variance across mean DNAm bins (e.g. a binned quantile variance filter, 10 bins of magnitude 0.1 or 10% DNAm, Supplementary Figure S7a). The recountmethylation Data Analyses vignette reproduces these analyses for two tissues, and the full analysis scripts are contained at https://github.com/metamaden/recountmethylationManuscriptSupplement/tree/main/inst/scripts/analyses.

### Statistical analyses and visualizations

Statistical analyses and visualizations were conducted with the R and Python programming languages. We used the numpy (v1.15.1), scipy (v1.1.0) and pandas v0.23.0 Python packages to manage jobs and downloads, perform data extraction and calculate summary statistics. We used the minfi v1.29.3 and limma v3.39.12 R/Bioconductor packages for downstream quality control, preprocessing and analyses. Plots were generated using base R functions, ggplot2 (v3.1.0) and ComplexHeatmap (v1.99.5) (41,42). To reproduce analyses, see Supplementary Methods, files at https://github.com/metamaden/recountmethylationManuscriptSupplement, and the Data Analyses vignette in the recountmethylation R/Bioconductor package.

### Supplemental Information

Supplemental Information, including methods, code, and scripts reproducing analyses, figures, and tables are accessible at https://github.com/metamaden/recountmethylationManuscriptSupplement. Large supplemental data files are accessible at https://recount.bio/data/recountmethylation_manuscript_supplement/.

### Companion R/Bioconductor package

Databases of the samples compiled and analyzed in this manuscript are accessible, along with comprehensive instructions and analysis examples, in the recountmethylation R/Bioconductor package at http://bioconductor.org/packages/devel/bioc/html/recountmethylation.html.

## RESULTS

### Recent growth in GEO DNAm array samples is linear

**Figure 1.**
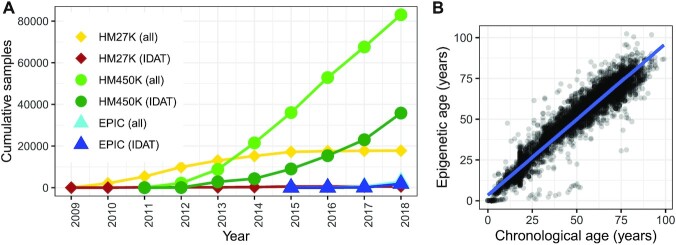
Cross-study summaries of DNAm array samples from GEO. (**A**) Cumulative samples by year using one of three major Illumina BeadArray DNAm array platforms (HM27K, HM450K and EPIC/HM850K, point shapes), showing either all samples or subsets with available IDAT files for each platform (line colors). Samples with IDATs using the HM450K platform (dark green line, circle shape) were compiled and analyzed (‘Materials and Methods’ and ‘Results’ sections). (**B**) Scatter plot of mined chronological (*x*-axis) and epigenetic (*y*-axis) ages, in years, with linear model fit (blue line), for 6019 non-cancer tissues run using the HM450K platform (‘Results’ section). Chronological age was mined from sample metadata. Epigenetic age was calculated using the model in (5) (‘Materials and Methods’ section).

We obtained sample IDATs and metadata for studies from the GEO. GEO is the largest public database for human DNAm array studies, and the majority of GEO’s DNAm array samples use one of three of Illumina’s BeadArray platforms: the HumanMethylation27K (HM27K), the HumanMethylation450K (HM450K) and EPIC, also known as the HumanMethylation850K (HM850K). On GEO, we identified 104 746 unique sample accession numbers (a.k.a. GSM IDs) from 1605 study accession numbers (a.k.a. GSE IDs) published using one of the three major Illumina DNAm array platforms (Figures 1A and Supplementary Figure S1). Among 1605 published studies, 74% used HM450K, 21% used HM27K and 5% used EPIC. Among 104 746 published samples, 79% were on HM450K, 18% on HM27K and 3% on EPIC. All three platforms showed increasing publication rates of samples and studies over the first three years of their availability. Few new studies and samples from 2013 to 2018 used the HM27K platform, while samples and studies using HM450K have grown linearly through 2018.

### Fewer than half of DNAm array studies on GEO include raw data

Raw data for a DNAm array sample is comprised of two IDAT files, one for each of the red and green color channels. Accessible raw data is important for uniform normalization of samples across studies, yet not all samples on GEO come with these data. In total, 37 919 samples (36% of total) included sample IDATs, where 93% were run on HM450K, 5% on EPIC, and 2% on HM27K. By platform, EPIC included the largest percentage of sample records with available IDATs at 63%, followed by HM450K at 43% and HM27K at just 3%. The more frequent availability of IDATs for newer arrays seems to reflect a significant shift in data submission norms well after the inception of the HM27K platform.

### Most annotated GEO HM450K samples with available raw data are from blood or brain

There were enough study and sample metadata for us to annotate 27 027 samples, 76% of the 35 360 we analyzed. We annotated these samples by applying regular expressions to the mined metadata. Our vocabulary for annotations was composed of 72 distinct terms (‘Materials and Methods’ section) strongly inspired by those used in the methylation array resource Marmal-aid (28). Tissue terms for blood accounted for the majority (18 212 samples, 67% of total), followed by brain (6690 samples, 25% of total), tumor (1977 samples, 7% of total), breast (1525 samples, 6% of total) and placenta (1338 samples, 5% of total). We further annotated disease and experiment group for 22 790 samples (64% of total) using 38 distinct disease- and group-related terms. Among these, disease terms for cancer were assigned to over half (13 131, 58% of total) of samples, while terms for normal, control, or healthy were assigned to 10 808 samples (47% of total). The most frequently annotated cancers included leukemia (2585 samples, 20% of total), breast cancer (511 samples, 4% of total), colorectal cancer (314 samples, 2% of total) and prostate cancer (196 samples, 1% of total). We compared disease and tissue characteristics to distinguish between tumor and normal samples from cancer patients, estimating that a third of samples were from tumor (‘Materials and Methods’ section and Supplementary Table 1).

### Chronological age is accurately predicted from epigenetic age in non-cancer tissues

Prior work showed chronological age can be predicted with high accuracy from DNAm among non-cancer tissues (5,6). We calculated model-based age predictions (a.k.a. ‘epigenetic ages’) from IDATs for 35 360 samples using the clock from (5), and we were able to mine chronological ages from metadata (a.k.a ‘chronological ages’) for a subset of 16 510 samples (47% of total, Supplementary Table and ‘Materials and Methods’ section). We investigated variance sources and differences between these ages, and determined whether missing chronological ages could be imputed using the epigenetic ages for certain types of samples.

In the 16 510 samples for which we were able to mine chronological ages from metadata, analysis of variance (ANOVA) showed most epigenetic age variation was explained by chronological age (52% of variances, *P* < 2.2e-16), followed by study (i.e. GSE ID; 24%, *P* < 2.2e-16), cancer status (7e-2%, *P* = 1.3e-9), and predicted sample type (8e-3%, *P* = 1.6e-2). Compared to variances attributed to the study variable, the relative low variances attributed to the cancer status and predicted sample type variables may be due to high study-specific variance in metadata completeness or availability. High age differences (12.9 years mean absolute difference, or MAD) and errors (R-squared = 0.76) likely resulted from either metadata inaccuracies, age label misattributions from our mining strategy, or inclusion of cancers and non-tissue samples (e.g. cell lines, ‘Materials and Methods’ section). In the subset of 6019 likely non-cancer tissue samples across 37 study records with study-wise MADs ≤ 10 years, epigenetic age variance contribution from mined age increased to 93% (ANOVA, *P* < 2.2e-16) and contribution from study decreased to 2% (*P* < 2.2e-16, Figure 1B). Unsurprisingly, the non-cancer tissue samples showed lower age differences (MAD = 4.5 years) and errors (R-squared = 0.94), and ages were highly correlated (Spearman Rho = 0.96, *P* < 2.2e-16), supporting the well-established finding that chronological age is accurately predicted from epigenetic age in non-cancer tissues (5,6). We therefore imputed missing chronological ages using the epigenetic age for non-cancer tissue analyses below.

We next studied age acceleration (5,6) by probing the differences between epigenetic and chronological ages among the 6019 previously identified samples with low study-wise age differences. Among the 68 samples with outlying positive age acceleration (≥15 years), the most frequently represented study accounted for 18 adipose samples from severely obese patients (accession ID: GSE61454 (43)). We observed 86 negative age acceleration outliers (≤−15 years), including 14 saliva samples from control subjects in a study of Parkinson’s disease (GSE111223 (44)) and 19 whole blood samples from patients with genetic syndromes (GSE97362 (45)). In the latter study, we suspect reported ages are inaccurate and older than actual ages (private correspondence, investigation ongoing).

### Almost a fifth of samples fail at least one of 17 BeadArray quality control assessments

Illumina prescribes 17 quality assessments for its 450K array, each measuring the performance of a different step in a methylation assay such as extension or hybridization (35,36). A given assessment comprises a quality metric and a minimum threshold value below which the assessment is failed. We call these assessments BeadArray controls. We used the 17 BeadArray controls and their minimum quality thresholds to evaluate assay qualities in 35 360 samples (Supplementary Tables S2 and S3). Results are summarized in Supplementary Figure 2A. The highest proportions of samples failed the non-polymorphic green and biotin staining green controls, with about 6.7% failing each (2381 and 2368 samples, respectively). By contrast, there are six BeadArray controls, each failed by fewer than 100 samples. A substantial number of samples (6813, 19% of total) failed at least one control. Of samples that failed at least one control, 4456 samples (66%) failed exactly one control, while 2357 samples (34%) failed more than one control. Of samples that failed more than one control, 634 failed both biotin staining controls and 648 failed both non-polymorphic controls. Samples failing at least one control were significantly enriched for certain labels including ‘cord blood,’ ‘brain cancer’, ‘prostate cancer’, ‘arthritis’ and ‘obese’ (binomial test, BH-adjusted *P* < 1e-3).

**Figure 2.**
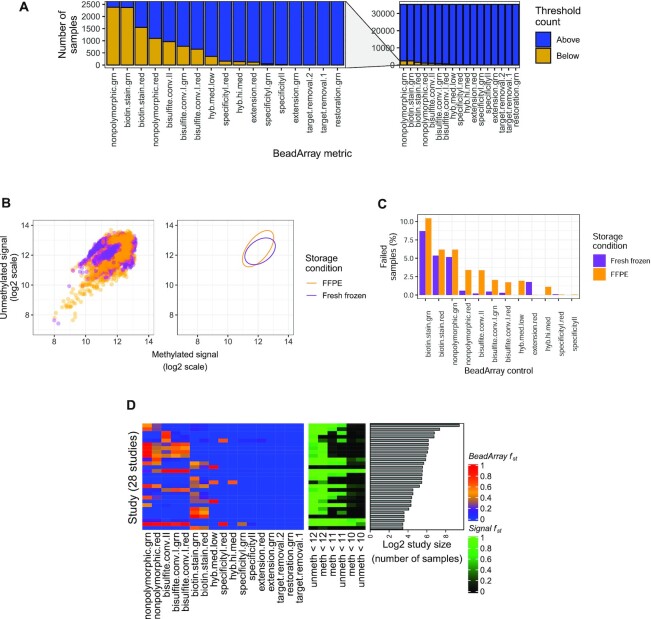
Quality analyses across samples, storage conditions, and studies. (**A**) Barplots counting samples (*y*-axis) falling above (blue) or below (gold) manufacturer-prescribed thresholds across the 17 BeadArray controls (*x*-axis). Full view is on right, and magnification is on left. (**B**) Scatter plots (left) and 95% confidence intervals (right) for *log*_2_ median methylated (*x*-axis) and *log*_2_ median unmethylated (*y*-axis) signal of 3467 formalin-fixed paraffin embedded (FFPE, orange) and 5729 fresh frozen samples (purple). (**C**) Percentages of FFPE (orange) and fresh frozen (purple) samples failing BeadArray controls. (**D**) Heatmaps depicting fraction (*f*_*st*_ in legends) of samples in a study failing quality assessments across 28 studies with high failure rates (*f*_*st*_ > 60%) and >10 samples. *BeadArray f*_*st*_ values are shown on the left, where blue is low, orange is intermediate and red is high. *Signal f*_*st*_ values for three methylated (M, ‘meth’) and unmethylated (U, ‘unmeth’) signal levels (10, 11 and 12) are shown in the middle, where black is low, dark green intermediate and light green is high. The log_2_ study sizes are shown on the right.

### The intrinsic dimension of the 17 BeadArray controls is small

We studied signals and outcomes to determine how best to use the BeadArray controls for sample quality assessments. Cross-sample signal distributions for five BeadArray controls were bimodal, with distinct low- and high-signal modes; minimum quality thresholds fell near low-signal modes (Supplementary Figure S3). For these controls, modifying minimum thresholds to more robustly capture low-signal samples could improve their utility. PCA of sample control performances showed the top five components explained 84% of overall variances. Component-wise ANOVAs showed that just five of the 17 controls explained the majority of sum of squared variances across these top five components (minimum = 67%, maximum = 99%, median = 97%). This suggests that the intrinsic dimension of sample quality is around 5. We conclude that sample quality is adequately captured by the performance of only 5 of the Illumina control probes (both biotin staining controls, both non-polymorphic controls and bisulfite conversion I red, Supplementary Figure S4).

### FFPE samples fail at least one BeadArray control almost twice as often as fresh frozen samples

We investigated the impact of storage conditions on sample quality across 28 studies by comparing 3467 formalin-fixed paraffin embedded (FFPE) and 5729 fresh frozen (FF) samples (Supplementary Table S4 and Figure 2B). FFPE samples showed greater variance than FF samples in both methylated (0.36 for FFPE versus 0.27 for FF) and unmethylated (0.50 for FFPE versus 0.21 for FF) signal channels. The trend could be driven either by condition-related sample characteristics (e.g. increased DNA deamination and/or lower DNA yield in FFPE, etc.) or differing preparation protocols (e.g. addition of the DNA restoration step for FFPE, (46–48)). Enriched labels also varied by storage condition among low-signal samples (binomial test, BH-adjusted *P* < 1e-3), where ‘colorectal,’ ‘intestine,’ and ‘mucosa’ were enriched among FFPE, while ‘nasal,’ ‘pancreas,’ and ‘epithelial’ were enriched among FF samples.

Across the 12 of 17 total BeadArray controls each with at least one failing sample, 228 FFPE samples (8.31% of total FFPE sample count) and 241 FF samples (4.21% of total FF sample count) failed more than one control. FFPE samples failed 10 of the 12 metrics between 0.1 and 3.2% more often than FF, including all three bisulfite conversion metrics (Figure 2C). FF samples had higher failure rates for two BeadArray controls (extension red and specificity I red; Supplementary Table S4). While no samples failed the restoration BeadArray control, increasing the minimum threshold for failure from the default manufacturer-prescribed value of 0 to 1, which is recommended as an alternative in Illumina documentation (35,36), failed 69 FFPE samples (2%) and one FF sample (2e-3%). In summary, while FFPE samples were of lower quality than FF samples across assessments, the differences were modest, and the vast majority of FFPE samples passed all controls considered.

### 10% of studies each have >60% samples failing quality assessments

Across 362 studies, we evaluated the fraction *f*_*st*_ of failed samples per study, defining a failed sample as one that either (i) fails at least one BeadArray control, or (ii) has log_2_ median methylated and log_2_ median unmethylated signals each <11 as described in (33) (Supplementary Table S5 and Supplementary Figure S5). Of the 36 studies each with $f_{st} >60\%$, samples fail in each of 23 studies due only to (i), samples fail in each of five studies due only to (ii), and samples fail in each of the remaining eight studies due to either (i) or (ii). These 36 studies ranged in size from 6 to 692 samples and comprised a total of 2020 samples, with a median study size of 23 samples. Of the 320 studies that remained after removing those with ≤10 samples, 28 showed $f_{st} >60\%$ (8.8% of remaining studies, Figure 2D). One of these was a study of condition-specific DNAm data reliability (GSE59038, (47)) and included several stress tests of the assay, so many failed samples are not unexpected. Another study was GSE62219 (49) and included blood from 10 young individuals. We further noted the previous study (50) also determined these samples were of low quality.

### DNAm principal component analysis shows clustering by tissue with greater variances among cancers

**Figure 3.**
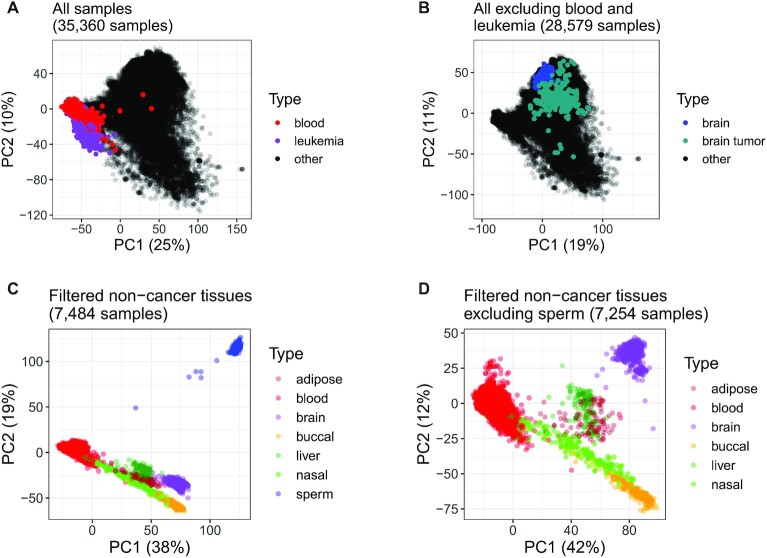
Scatter plots of top two components from PCAs of autosomal DNAm (‘Materials and Methods’ section). Each axis label also contains percent of total variance explained by the component. (**A**) PCA of 35 360 samples, with color labels for non-cancer blood (*N* = 6001 samples, red points) and leukemias (780, purple) and remaining samples (28 579, black). (**B**) PCA of 28 579 samples remaining after exclusion of blood and leukemias from (A), highlighting non-cancer brain (*N* = 602 samples, blue), brain tumors (221, dark cyan) and remaining samples (27 756, black points). Facet plots of sample subsets in (A) and (B) are shown in Supplementary Figure S6. (**C**) and (**D**) display samples from seven non-cancer tissues for which at least 100 samples were available from at least two studies (‘Materials and Methods’ section). (C) PCA of 7484 samples from all seven tissue types, including sperm (*N* = 230 samples, blue), adipose (104, dark red), blood (6,001, red), brain (602, purple), buccal (244, orange), nasal (191, light green) and liver (112, dark green). (D) PCA of 7254 non-cancer tissue samples remaining from (C) after exclusion of sperm, with color labels as in (C).

We studied DNAm variance using PCAs of autosomal DNAm (Figure 3) as measured by noob-normalized Beta-values (‘Materials and Methods’ section). The first two components from PCA of 35 360 samples explained 35% of total variance, with PC1’s contribution 25% and PC2’s contribution 10% (Figure 3A). Four outlying blood samples (PC1 > −10) included two from whole blood, one of T cells, and one stem cell sample from umbilical cord blood (left plot of Supplementary Figure S6a). For the top two components, leukemia samples showed greater variances than blood samples: the ratio of variance in PC1 for leukemia samples to variance in PC1 for blood samples was 1.25 (F-test *P* < 1e-2), and the ratio of variance in PC2 for leukemia samples to variance in PC2 in blood samples was 6.18 (F-test *P* < 1e-2). This is consistent with how (i) leukemia samples have greater variance than blood samples at each of the majority of probes (311 127 or 66%) and (ii) leukemia samples have greater median variance than blood samples across probes (median Beta-value variance for blood samples = 1e-3, median Beta-value variance for leukemia samples = 5e-3; Supplementary Figure S6b).

From PCA of the 28 579 samples remaining after blood and leukemia samples were removed (Figure 3B), the first two components explained 30% of total variance, with PC1’s contribution 19% and PC2’s contribution 11%. Seven outlying (PC1 > 0, PC2 < -5) brain tumor samples included two primary tumors and one metastasis each from medulloblastoma cases, as well as four brain metastases from uncertain primary tumors, from the studies GSE108576 (51,52) and GSE63669 (53) (Supplementary Figure S6c). For the top two components, brain tumors showed greater variances than non-cancer brain samples: the ratio of variance in PC1 for brain tumors to variance in PC1 for non-cancer brain samples was 12.05 (F-test *P* < 1e-5), and the ratio of variance in PC2 for brain tumors to the variance in PC2 for non-cancer brain samples was 22.40 (F-test *P* < 1e-5). This is consistent with how (i) brain tumors have greater variance than non-cancer brain samples at each of the majority of probes (444 304, or 94%), and (ii) brain tumors have greater median variance than non-cancer brain samples across probes (median Beta-value variance for non-cancer brain samples = 1e-3, median Beta-value variance for brain tumors = 1e-2; Supplementary Figure S6d). Our findings are consistent with previous evidence of higher DNAm variances in cancers compared to non-cancer samples (54,55).

PCA of 7484 samples from seven non-cancer tissues (adipose, nasal, blood, brain, buccal, sperm and liver), which we also used to study DNAm variability (below), showed clear clustering by tissue. The first two components explained 57% of total variance, with PC1’s contribution 38% and PC2’s contribution 19%. Sperm samples clustered far apart from the six somatic tissues (Figure 3C). After repeating PCA with sperm samples excluded, the first two components still explained over half (54%) of total variance, with PC1’s contribution 42% and PC2’s contribution 12% (Figure 3D).

### Over two-thirds of CpG probes that do not distinguish tissues map to gene promoters near CpG islands

CpG probes with low DNAm variation and low mean DNAm differences across experimental groups are less informative for quantifying group-specific DNAm differences. We analyzed autosomal DNAm variation in seven distinct tissues (adipose, nasal, blood, brain, buccal, sperm and liver), as measured by noob-normalized, study-corrected Beta-values (‘Materials and Methods’ section). We identified 4577 probes each with consistently low variance (≤10th quantile) in each tissue and low difference between highest and lowest mean Beta-value (<0.01) across tissues (Supplementary Figures S7a and S9 as well as Supplementary Table S7). Among probes with consistently low variance, 4111 (90% of total) mapped to genes in CpG islands, typically at promoter regions of CpG island-overlapping genes (2203 probes) and these fractions represented significant increases compared to the background of all autosomal CpG probes (binomial tests, *P*-values < 1e-3). It is likely the 4577 probes are of low utility for quantifying DNAm differences across tissues, and their removal prior to performing an EWAS across non-cancer tissues could help increase statistical power.

### Over two-thirds of CpG probes that distinguish tissues map to genes

**Figure 4.**
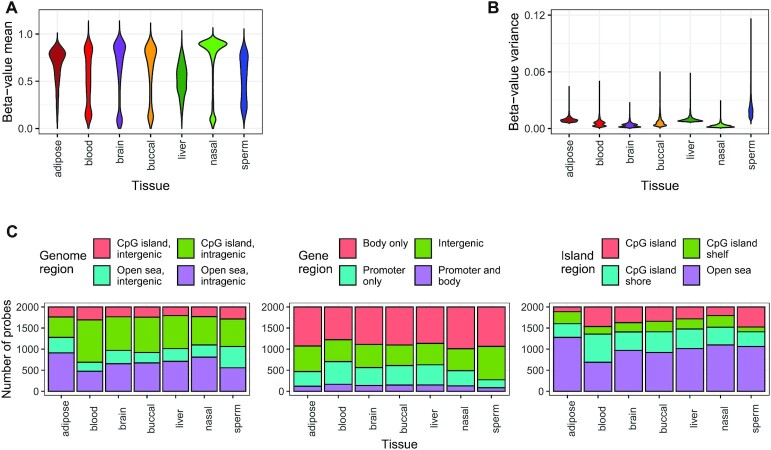
DNAm and genome mapping patterns among 14 000 CpG probes showing tissue-specific high variance in seven tissues (2000 probes per tissue, tissues: adipose, blood, brain, buccal, liver, nasal and sperm). (**A** and **B**) Violin plots of (A) means and (B) variances of normalized Beta-values across tissue-specific probes. (**C**) Stacked barplots of genome region mappings (number of CpG probes, *y*-axis) across tissue-specific probes (*x*-axis). Color fills depict (left) island and gene overlap, (center) gene region overlap and (right) island region overlap.

We identified 2000 CpG probes in each of seven distinct non-cancer tissues (adipose, nasal, blood, brain, buccal, sperm and liver) with high and tissue-specific variation in autosomal DNAm, as measured by noob-normalized, study-corrected Beta-values (‘Materials and Methods’ section, Supplementary Table S8, and Figures 4 and Supplementary S7a). Distinctive patterns in DNAm across these probe sets may point to tissue-specific factors, such as differences in environment exposure, cellular signaling and cell division rates. Compared to the background of all autosomal CpG probes, adipose and sperm had significantly lower fractions of gene-mapping probes, and all tissues except for blood had significantly greater fractions of both open sea-mapping probes and gene body-mapping probes (binomial tests, BH-adjusted *P* < 1e-3). Of the 14 000 total high-variance probes, 10 016 (71%) mapped to a gene region, typically at the gene body (8006 probes, Supplementary Table S8). The highest mean Beta-values were observed for nasal and adipose tissues (Figure 4A), and the highest variances were observed for sperm and adipose tissues (Figure 4B), while probes in blood had relative low means and variances. While most probes mapped to open seas in liver (1014 probes), nasal (1100), adipose (1280) and sperm (1063), greater fractions of open sea probes mapped to genes in liver (70% of open sea probes), nasal (74%) and adipose (71%) than in sperm (52%, Figure 4C). This observed sparsity of CpG island regions and enrichment of open sea, intergenic and gene body regions among CpG probes with tissue-specific DNAm was recently corroborated in an independent study comparing DNAm in matched sperm and blood samples directly (30). This corroboration was especially striking because the discovery set samples in (30) were processed on the newer EPIC/HM850K rather than the HM450K platform.

### Normalized Beta-values for GEO DNAm array studies are rapidly accessed via the recountmethylation package

To accommodate a wide range of analysis strategies, DNAm assays and sample metadata were compiled into databases in two distinct formats, including Hierarchical Data Format 5 (HDF5) and HDF5-SummarizedExperiment. HDF5-SummarizedExperiment compilations are tailored for rapidly executing data summaries and query operations in the R/Bioconductor framework via DelayedArray objects. Raw red and green signals are provided as HDF5 (120 GB) and HDF5-SummarizedExperiment (119 GB) files and raw methylated and unmethylated signals and noob-normalized Beta-values are provided as HDF5-SummarizedExperiment files (94 and 133 GB, respectively). The recountmethylation R/Bioconductor package facilitates database access as described in the user’s manual. It allows full database utilization with rapid queries on the provided sample metadata, including model-based estimates for sex, epigenetic age and blood cell types (5,33,34). The package Data Analyses vignette further provides code to reproduce our comparisons of mined and epigenetic ages, sample storage type quality comparisons and tissue-specific DNAm variability analyses described above.

## DISCUSSION

### Limitations of this study

We conducted a cross-study analysis of methylation array samples comprising a large subset of available HM450K samples on GEO. While we omitted studies using the HM27K and EPIC platforms, our compilation strategy could also be generalized to these platforms (‘Materials and Methods’ section, below). Further, while our results suggest BeadArray controls could be improved by applying different quantitative thresholds for failure, it remains unclear whether a single universal threshold or multiple experiment-specific thresholds is desirable for each (‘Results’ section, Supplementary Figure S3). Nonetheless, five of 17 BeadArray controls (both Biotin Staining controls, both non-polymorphic controls, and Bisulfite Conversion I Red) are demonstrably useful for assessing the quality of an experiment. This finding also means a stringent quality threshold of ≥1 failed controls, which we used for cross-study analyses, mainly filtered samples due to failure in at least one of these five principal controls. We further lacked a definitive gold standard set of well-described DNAm array samples, which could allow for more detailed estimations of metadata errors beyond direct concordances, or for more informative assessments of quality metric behaviors. Our metadata mapping and annotation strategy can also be improved to better capture available metadata for certain samples, such as gestational and maternal ages for placenta samples (‘Materials and Methods’ section). Finally, our DNAm variability study across seven distinct non-cancer tissues used a within-tissue preprocessing approach (‘Materials and Methods’ section). We were constrained this way because study-specific variation was high relative to tissue-specific variation, and effective study-and-tissue normalization would have required considerably more data from studies of multiple tissues than were available at the time of analysis.

### Recommendations for metadata reporting

Thoroughly characterizing samples submitted to public archives with accurate metadata makes them easier to repurpose for new studies. After manually inspecting hundreds of DNAm array studies on GEO, we formulated some best practices for the submitter who is labeling samples in a study to facilitate their discoverability and improve their utility for other investigators:

- Include key attributes (sex, age, tissue, disease, etc.) even when any one is the same across a sample set, since that attribute may vary in a cross-study analysis.
- Repeat study-level metadata in sample-level metadata. This includes sample types (e.g. tissue, cell line, etc.) and characteristics (e.g. storage conditions, preparation steps, etc.).
- Include units of numerical variables to ensure their proper interpretation.
- Clarify circumstances under which attributes were obtained where appropriate. For example, age *at diagnosis*, *tumor-adjacent* normal tissue and blood *from leukemia patient*.

### Next steps

We have several methodological changes planned that will improve the DNAm array databases accessible with the recountmethylation R/Bioconductor package. First, future compilations will add samples run on the newer EPIC/HM850K platform, allowing for novel cross-platform analyses. Further, our metadata handling pipeline will be revised to be fully automated by using regular expressions to recognize key metadata. This will replace the manual variable aggregation step (‘Materials and Methods’ section). Finally, we will support regular compilation updates by enabling rapid setup and better dependency handling (e.g. with virtual environments). These improvements will empower the researcher to maintain a comprehensive compilation of DNAm array IDATs from GEO in a wide array of computing environments.

### Concluding remarks

We performed extensive analyses of 35 360 HM450K samples with IDATs from 362 studies in GEO, approximately three times the number of samples considered in prior cross-study analyses (5,28–30). We further released the R/Bioconductor package recountmethylation including our new tissue and disease state labels, model-based predictions for age, sex and blood cell composition, as well as noob-normalized Beta-values for array samples. This resource should prove valuable for reusing publicly available methylation data.

## Supplementary Material

lqab025_Supplemental_Files

## References

[1] Feinberg A.P., Tycko B. The history of cancer epigenetics. Nat. Rev. Cancer. 2004; 4:143–153. 14732866 10.1038/nrc1279

[2] Ziller M.J., Gu H., Müller F., Donaghey J., Tsai L.T.-Y., Kohlbacher O., De Jager P.L., Rosen E.D., Bennett D.A., Bernstein B.E. et al. Charting a dynamic DNA methylation landscape of the human genome. Nature. 2013; 500:477–481. 23925113 10.1038/nature12433 PMC3821869

[3] Lokk K., Modhukur V., Rajashekar B., Märtens K., Mägi R., Kolde R., Koltšina M., Nilsson T.K., Vilo J., Salumets A. et al. DNA methylome profiling of human tissues identifies global and tissue-specific methylation patterns. Genome Biol. 2014; 15:r54. 24690455 10.1186/gb-2014-15-4-r54 PMC4053947

[4] Jones P.A. Functions of DNA methylation: islands, start sites, gene bodies and beyond. Nat. Rev. Genet. 2012; 13:484–492. 22641018 10.1038/nrg3230

[5] Horvath S. DNA methylation age of human tissues and cell types. Genome Biol. 2013; 14:R115. 24138928 10.1186/gb-2013-14-10-r115 PMC4015143

[6] Hannum G., Guinney J., Zhao L., Zhang L., Hughes G., Sadda S., KlIotzle B., Bibikova M., Fan J.-B., Gao Y. et al. Genome-wide methylation profiles reveal quantitative views of human aging rates. Mol. Cell. 2013; 49:359–367. 23177740 10.1016/j.molcel.2012.10.016 PMC3780611

[7] Yang Z., Wong A., Kuh D., Paul D.S., Rakyan V.K., Leslie R.D., Zheng S.C., Widschwendter M., Beck S., Teschendorff A.E. Correlation of an epigenetic mitotic clock with cancer risk. Genome Biol. 2016; 17:205. 27716309 10.1186/s13059-016-1064-3 PMC5046977

[8] Byun H.-M., Siegmund K.D., Pan F., Weisenberger D.J., Kanel G., Laird P.W., Yang A.S. Epigenetic profiling of somatic tissues from human autopsy specimens identifies tissue- and individual-specific DNA methylation patterns. Hum. Mol. Genet. 2009; 18:4808–4817. 19776032 10.1093/hmg/ddp445 PMC4481584

[9] Bell J.T., Pai A.A., Pickrell J.K., Gaffney D.J., Pique-Regi R., Degner J.F., Gilad Y., Pritchard J.K. DNA methylation patterns associate with genetic and gene expression variation in HapMap cell lines. Genome Biol. 2011; 12:R10. 21251332 10.1186/gb-2011-12-1-r10 PMC3091299

[10] Heyn H., Vidal E., Ferreira H.J., Vizoso M., Sayols S., Gomez A., Moran S., Boque-Sastre R., Guil S., Martinez-Cardus A. et al. Epigenomic analysis detects aberrant super-enhancer DNA methylation in human cancer. Genome Biol. 2016; 17:11. 26813288 10.1186/s13059-016-0879-2 PMC4728783

[11] Issa J.-P. CpG island methylator phenotype in cancer. Nat. Rev. Cancer. 2004; 4:988–993. 15573120 10.1038/nrc1507

[12] Irizarry R.A., Ladd-Acosta C., Wen B., Wu Z., Montano C., Onyango P., Cui H., Gabo K., Rongione M., Webster M. et al. The human colon cancer methylome shows similar hypo- and hypermethylation at conserved tissue-specific CpG island shores. Nat. Genet. 2009; 41:178–186. 19151715 10.1038/ng.298 PMC2729128

[13] Wang Z., Yin J., Zhou W., Bai J., Xie Y., Xu K., Zheng X., Xiao J., Zhou L., Qi X. et al. Complex impact of DNA methylation on transcriptional dysregulation across 22 human cancer types. Nucleic Acids Res. 2020; 48:2287–2302. 32002550 10.1093/nar/gkaa041 PMC7049702

[14] Peters T.J., Buckley M.J., Statham A.L., Pidsley R., Samaras K., V Lord R., Clark S.J., Molloy P.L. *De novo* identification of differentially methylated regions in the human genome. Epigenet. Chromatin. 2015; 8:6. 10.1186/1756-8935-8-6 PMC4429355 25972926

[15] Siegmund K.D. Statistical approaches for the analysis of DNA methylation microarray data. Hum. Genet. 2011; 129:585–595. 21519831 10.1007/s00439-011-0993-x PMC3166559

[16] Michels K.B., Binder A.M., Dedeurwaerder S., Epstein C.B., Greally J.M., Gut I., Houseman E.A., Izzi B., Kelsey K.T., Meissner A. et al. Recommendations for the design and analysis of epigenome-wide association studies. Nat. Methods. 2013; 10:949–955. 24076989 10.1038/nmeth.2632

[17] Edgar R., Domrachev M., Lash A.E. Gene Expression Omnibus: NCBI gene expression and hybridization array data repository. Nucleic Acids Res. 2002; 30:207–210. 11752295 10.1093/nar/30.1.207 PMC99122

[18] Barrett T., Wilhite S.E., Ledoux P., Evangelista C., Kim I.F., Tomashevsky M., Marshall K.A., Phillippy K.H., Sherman P.M., Holko M. et al. NCBI GEO: archive for functional genomics data sets—update. Nucleic Acids Res. 2012; 41:D991–D995. 23193258 10.1093/nar/gks1193 PMC3531084

[19] Athar A., Füllgrabe A., George N., Iqbal H., Huerta L., Ali A., Snow C., Fonseca N.A., Petryszak R., Papatheodorou I. et al. ArrayExpress update—from bulk to single-cell expression data. Nucleic Acids Res. 2019; 47:D711–D715. 30357387 10.1093/nar/gky964 PMC6323929

[20] Weinstein J.N., Collisson E.A., Mills G.B., Shaw K.R.M., Ozenberger B.A., Ellrott K., Shmulevich I., Sander C., Stuart J.M., Network C.G.A.R. et al. The cancer genome atlas pan-cancer analysis project. Nat. Genet. 2013; 45:1113–1120. 24071849 10.1038/ng.2764 PMC3919969

[21] Dunham I., Kundaje A., Aldred S.F., Collins P.J., Davis C.A., Doyle F., Epstein C.B., Frietze S., Harrow J., Kaul R. et al. An integrated encyclopedia of DNA elements in the human genome. Nature. 2012; 489:57–74. 22955616 10.1038/nature11247 PMC3439153

[22] Davis C.A., Hitz B.C., Sloan C.A., Chan E.T., Davidson J.M., Gabdank I., Hilton J.A., Jain K., Baymuradov U.K., Narayanan A.K. et al. The Encyclopedia of DNA elements (ENCODE): data portal update. Nucleic Acids Res. 2018; 46:D794–D801. 29126249 10.1093/nar/gkx1081 PMC5753278

[23] Li M., Zou D., Li Z., Gao R., Sang J., Zhang Y., Li R., Xia L., Zhang T., Niu G. et al. EWAS Atlas: a curated knowledgebase of epigenome-wide association studies. Nucleic Acids Res. 2019; 47:D983–D988. 30364969 10.1093/nar/gky1027 PMC6324068

[24] Xiong Z., Li M., Yang F., Ma Y., Sang J., Li R., Li Z., Zhang Z., Bao Y. EWAS Data Hub: a resource of DNA methylation array data and metadata. Nucleic Acids Res. 2019; 48:D890–D895. 10.1093/nar/gkz840 PMC6943079 31584095

[25] Liu D., Zhao L., Wang Z., Zhou X., Fan X., Li Y., Xu J., Hu S., Niu M., Song X. et al. EWASdb: epigenome-wide association study database. Nucleic Acids Res. 2019; 47:D989–D993. 30321400 10.1093/nar/gky942 PMC6323898

[26] Li R., Liang F., Li M., Zou D., Sun S., Zhao Y., Zhao W., Bao Y., Xiao J., Zhang Z. MethBank 3.0: a database of DNA methylomes across a variety of species. Nucleic Acids Res. 2018; 46:D288–D295. 29161430 10.1093/nar/gkx1139 PMC5753180

[27] Xiong Y., Wei Y., Gu Y., Zhang S., Lyu J., Zhang B., Chen C., Zhu J., Wang Y., Liu H. et al. DiseaseMeth version 2.0: a major expansion and update of the human disease methylation database. Nucleic Acids Res. 2017; 45:D888–D895. 27899673 10.1093/nar/gkw1123 PMC5210584

[28] Lowe R., Rakyan V.K. Marmal-aid—a database for Infinium HumanMethylation450. BMC Bioinformatics. 2013; 14:359. 24330312 10.1186/1471-2105-14-359 PMC3878775

[29] Heiss J.A., Just A.C. Identifying mislabeled and contaminated DNA methylation microarray data: an extended quality control toolset with examples from GEO. Clin. Epigenet. 2018; 10:73. 10.1186/s13148-018-0504-1 PMC5984806 29881472

[30] Åsenius F., Gorrie-Stone T.J., Brew A., Panchbhaya Y., Williamson E., Schalkwyk L.C., Rakyan V.K., Holland M.L., Marzi S.J., Williams D.J. The DNA methylome of human sperm is distinct from blood with little evidence for tissue-consistent obesity associations. PLOS Genet. 2020; 16:e1009035. 33048947 10.1371/journal.pgen.1009035 PMC7584170

[31] Huber W., Carey V.J., Gentleman R., Anders S., Carlson M., Carvalho B.S., Bravo H.C., Davis S., Gatto L., Girke T. et al. Orchestrating high-throughput genomic analysis with Bioconductor. Nat. Methods. 2015; 12:115–121. 25633503 10.1038/nmeth.3252 PMC4509590

[32] Triche T.J., Weisenberger D.J., Van Den Berg D., Laird P.W., Siegmund K.D. Low-level processing of Illumina Infinium DNA Methylation BeadArrays. Nucleic Acids Res. 2013; 41:e90. 23476028 10.1093/nar/gkt090 PMC3627582

[33] Aryee M.J., Jaffe A.E., Corrada-Bravo H., Ladd-Acosta C., Feinberg A.P., Hansen K.D., Irizarry R.A. Minfi: a flexible and comprehensive Bioconductor package for the analysis of Infinium DNA methylation microarrays. Bioinformatics. 2014; 30:1363–1369. 24478339 10.1093/bioinformatics/btu049 PMC4016708

[34] Houseman E., Accomando W.P., Koestler D.C., Christensen B.C., Marsit C.J., Nelson H.H., Wiencke J.K., Kelsey K.T. DNA methylation arrays as surrogate measures of cell mixture distribution. BMC Bioinformatics. 2012; 13:86. 22568884 10.1186/1471-2105-13-86 PMC3532182

[35] Illumina Illumina Genome Studio Methylation Module v1.8. 2010;

[36] Illumina BeadArray Controls Reporter Software Guide. 2015;

[37] Bernstein M.N., Doan A., Dewey C.N., Wren J. MetaSRA: normalized human sample-specific metadata for the Sequence Read Archive. Bioinformatics. 2017; 33:2914–2923. 28535296 10.1093/bioinformatics/btx334 PMC5870770

[38] Malladi V.S., Erickson D.T., Podduturi N.R., Rowe L.D., Chan E.T., Davidson J.M., Hitz B.C., Ho M., Lee B.T., Miyasato S. et al. Ontology application and use at the ENCODE DCC. Database. 2015; 2015:bav010. 25776021 10.1093/database/bav010 PMC4360730

[39] Weinberger K., Dasgupta A., Attenberg J., Langford J., Smola A. Feature Hashing for Large Scale Multitask Learning. 2010; arXiv doi:27 February 2010, preprint: not peer reviewed https://arxiv.org/abs/0902.2206.

[40] Kane D.M., Nelson J. Sparser johnson-lindenstrauss transforms. JACM. 2014; 61:4.

[41] Gu Z., Eils R., Schlesner M. Complex heatmaps reveal patterns and correlations in multidimensional genomic data. Bioinformatics. 2016; 32:2847–2849. 27207943 10.1093/bioinformatics/btw313

[42] Wickham H. ggplot2: Elegant Graphics for Data Analysis. 2016; NY Springer.

[43] Bonder M.J., Kasela S., Kals M., Tamm R., Lokk K., Barragan I., Buurman W.A., Deelen P., Greve J.-W., Ivanov M. et al. Genetic and epigenetic regulation of gene expression in fetal and adult human livers. BMC Genomics. 2014; 15:860. 25282492 10.1186/1471-2164-15-860 PMC4287518

[44] Horvath S., Ritz B.R. Increased epigenetic age and granulocyte counts in the blood of Parkinson’s disease patients. Aging. 2015; 7:1130–1142. 26655927 10.18632/aging.100859 PMC4712337

[45] Butcher D.T., Cytrynbaum C., Turinsky A.L., Siu M.T., Inbar-Feigenberg M., Mendoza-Londono R., Chitayat D., Walker S., Machado J., Caluseriu O. et al. CHARGE and Kabuki Syndromes: gene-specific DNA methylation signatures identify epigenetic mechanisms linking these clinically overlapping conditions. Am. J. Hum. Genet. 2017; 100:773–788. 28475860 10.1016/j.ajhg.2017.04.004 PMC5420353

[46] Espinal A.C., Wang D., Yan L., Liu S., Tang L., Hu Q., Morrison C.D., Ambrosone C.B., Higgins M.J., Sucheston-Campbell L.E. A methodological study of genome-wide DNA methylation analyses using matched archival formalin-fixed paraffin embedded and fresh frozen breast tumors. Oncotarget. 2017; 8:14821–14829. 28118602 10.18632/oncotarget.14739 PMC5362446

[47] Siegel E.M., Berglund A.E., Riggs B.M., Eschrich S.A., Putney R.M., Ajidahun A.O., Coppola D., Shibata D. Expanding epigenomics to archived FFPE tissues: an evaluation of DNA repair methodologies. Cancer Epidemiol. Prevent. Biomark. 2014; 23:2622–2631. 10.1158/1055-9965.EPI-14-0464 PMC4256717 25472669

[48] Kling T., Wenger A., Beck S., Carén H. Validation of the MethylationEPIC BeadChip for fresh-frozen and formalin-fixed paraffin-embedded tumours. Clin. Epigenet. 2017; 9:33. 10.1186/s13148-017-0333-7 PMC5379646 28392843

[49] Acevedo N., Reinius L.E., Vitezic M., Fortino V., Söderhäll C., Honkanen H., Veijola R., Simell O., Toppari J., Ilonen J. et al. Age-associated DNA methylation changes in immune genes, histone modifiers and chromatin remodeling factors within 5years after birth in human blood leukocytes. Clin. Epigenet. 2015; 7:34. 10.1186/s13148-015-0064-6 PMC4396570 25874017

[50] Sharp G.C., Ho K., Davies A., Stergiakouli E., Humphries K., McArdle W., Sandy J., Davey Smith G., Lewis S.J., Relton C.L. Distinct DNA methylation profiles in subtypes of orofacial cleft. Clin. Epigenet. 2017; 9:63. 10.1186/s13148-017-0362-2 PMC5465456 28603561

[51] Orozco J.I., Manughian-Peter A.O., Salomon M.P., Marzese D.M. Epigenetic classifiers for precision diagnosis of brain tumors. Epigenet. Insights. 2019; 12:2516865719840284. 30968063 10.1177/2516865719840284 PMC6444760

[52] Salomon M.P., Orozco J.I., Wilmott J.S., Hothi P., Manughian-Peter A.O., Cobbs C.S., Scolyer R.A., Hoon D.S., Marzese D.M. Brain metastasis DNA methylomes, a novel resource for the identification of biological and clinical features. Sci. Data. 2018; 5:180245. 30398472 10.1038/sdata.2018.245 PMC6219670

[53] Wang X., Dubuc A.M., Ramaswamy V., Mack S., Gendoo D.M.A., Remke M., Wu X., Garzia L., Luu B., Cavalli F. et al. Medulloblastoma subgroups remain stable across primary and metastatic compartments. Acta Neuropathol. 2015; 129:449–457. 25689980 10.1007/s00401-015-1389-0 PMC4333718

[54] Hansen K.D., Timp W., Bravo H.C., Sabunciyan S., Langmead B., McDonald O.G., Wen B., Wu H., Liu Y., Diep D. et al. Increased methylation variation in epigenetic domains across cancer types. Nat. Genet. 2011; 43:768–775. 21706001 10.1038/ng.865 PMC3145050

[55] Teschendorff A.E., Jones A., Fiegl H., Sargent A., Zhuang J.J., Kitchener H.C., Widschwendter M. Epigenetic variability in cells of normal cytology is associated with the risk of future morphological transformation. Genome Med. 2012; 4:24. 22453031 10.1186/gm323 PMC3446274

